# Description of the draft genome sequence of *Streptococcus salivarius* eK12, a derivative of the probiotic strain *Streptococcus salivarius* K12 with a modified plasmid

**DOI:** 10.1128/mra.00355-25

**Published:** 2025-07-08

**Authors:** Francesco Di Pierro, Aswin Thacharodi, Muthiah Kumaraswami, Alexander Bertuccioli, Maria Laura Tanda, Nicola Zerbinati

**Affiliations:** 1 Microbiota International Clinical Society, Torino, Italy; 2 Scientific & Research Department, Velleja Research, Milano, Italy; 3 Department of Medicine and Technological Innovation, University of Insubria 19045, Varese, Italy; 4 Center for Molecular and Translational Human Infectious Diseases Research, Houston Methodist Research Institute 167626 , Houston, Texas, USA; 5 Department of Pathology and Genomic Medicine, Houston Methodist Hospital 23534 , Houston, Texas, USA; 6 Department of Biomolecular Sciences, University of Urbino Carlo Bo 204212 , Urbino, Italy; 7 Endocrine Unit, Department of Medicine and Surgery, University of Insubria, Varese, Italy; University of Maryland School of Medicine, Baltimore, Maryland, USA

**Keywords:** engineered probiotic, salivabactin, speB

## Abstract

The draft genome of *Streptococcus salivarius* eK12, a derivative of the strain K12 with a modified plasmid (pRSSL1), is presented. eK12 was engineered to enhance its probiotic properties against *S. pyogenes* (GAS) by modulating salivabactin production and interfering with a GAS quorum sensing pathway.

## ANNOUNCEMENT

*Streptococcus salivarius* K12 is a probiotic isolated in 1989 from a child’s tongue on Mitis Salivarius agar (Difco Laboratories, Detroit) (1, 2). To potentiate it against *Streptococcus pyogenes* (GAS), the derivative strain eK12 (LMG P-33696) was engineered by modifying pRSSL1 (3, 4). A ~600 bp DNA fragment flanking the gene of interest was amplified with pJL nip A: CAATACATTAAGTGTGGAGGTAACTATTAGTGGTTGATTTTACTATTTCTTTGA 5′ and pJL nip B: TCAAAGAAATAGTAAAATCAACCACTAATAGTTACCTCCACACTTAATGTATTG 3′ primers and cloned into pJL1055. It was introduced into K12 by competence-based uptake using ComS peptide (LPYFAGCL, 5 µg plasmid). Antibiotic-resistant colonies were selected, plasmid-cured, and verified by sequencing. Mutation of the *nip* start to stop codon was introduced using primers pJL nip* A/B. The native promoter of the *sar*BGC was replaced with the constitutive *PtufA* promoter, resulting in elevated *sar* gene expression and enhanced salivabactin production.

K12 strain was obtained from American Type Culture Collection (ATCC) (BAA-1024). Colonies were cultured in Todd Hewitt broth (BD, USA) with 1% yeast extract (Gibco, Thermo Fisher, USA). DNA was extracted by the Qiagen DNeasy Blood and Tissue Kit (Qiagen, Germany) and quantified by the Qubit 2.0 Fluorometer (Thermo Fisher, USA). Libraries were prepared with the NEBNext Ultra II Kit (NEB, USA) and sequenced by Azenta Life Sciences (USA) by Illumina (best match accession number ALIF01000001; NCBI).

Antibiotic resistance was tested by the Clinical and Laboratory Standards Institute (CLSI) method (5) and evaluated per European Food Safety Authority (EFSA) guidelines, showing no phenotypic resistance (Table 1).

**TABLE 1:** MIC values (µg/mL) comparison between *S. salivarius* eK12 and the parental strain^*a*^^,^^*b*^

Drug	MIC value (µg/mL)		
	*S. salivarius* eK12	*S. salivarius* K12	EFSA cutoff
Gentamicin	8	8	32
Kanamycin	64	64	NR
Streptomycin	32	32	64
Neomycin	16	32	
Tetracycline	1	1	4
Erythromycin	0.12	0.12	2
Clindamycin	0.06	0.06	2
Chloramphenicol	2	1	4
Ampicillin	0.12	0.12	2
Penicillin	0.06	0.06	
Vancomycin	0.5	0.5	4
Quinupristina-dalfopristina	0.5	1	
Linezolid	1	1	
Trimethoprim	16	32	
Ciprofloxacin	4	4	NR
Rifampicin	<0.12	<0.12	

^*a*^ NR: not requested.

^*b*^ Empty cells indicates that the EFSA never established a cutuff value.

Sequencing was performed by Illumina NovaSeq X Plus with 2 × 150 bp reads, generating around 2.4 million reads (331 million bases). The BacFlux v1.1.8 workflow was used for data processing, following the stated parameters (6). Raw reads were quality filtered and adapter trimmed using fastp v0.23.4, yielding 2.2 million high-quality sequences (91%) with an average Phred score of Q30 (7). These reads were assembled into contigs with SPAdes v4.0.0 (8). Contigs shorter than 500 bp or with coverage below 2× were discarded. Assembly quality was assessed with QualiMap v2.3, showing a mean coverage of 127× and 39.73% GC content (9). The assembly included 34 contigs, totaling 2,354,147 bp, and an N50 of 273,236 bp, with the largest contig being 672,788 bp, as assessed by Quast v5.2.0 (10). Completeness was 99.39%, as assessed by CheckM v1.2.3 (11). Potential contaminants were identified with BlobTools v1.1.1, but no significant contamination was found (12). Phylogenetic analysis confirmed the strain as *S. salivarius* (sp001556435) using GTDB-Tk v2.4.0 (13, 14). ABRicate v1.0.1 did not detect any acquired antibiotic resistance genes (15), and mapping against the CARD database using BBMap v39.06 showed no concerning AMR genes (16, 17). Viral gene presence was checked with Virsorter2 and CheckV (18, 19), revealing a plasmid contig with proviral genes, as identified by Platon v1.7 and PGAP v6.9 (20). Comparison with the *S. salivarius* K12 reference genome (NCBI PRJNA168659) using BLASTv2.15.0 and BBMap v39.06 confirmed that the eK12 strain only differs by an engineered insertion into plasmid pRSSL1, including the inactivation of the *nip* gene and the introduction of a 235 bp *PtufA* promoter (21, 22). No other genomic differences were found with Snippy v4.6.0 (23).

## Data Availability

The whole-genome shotgun project has been deposited in NCBI under the BioProject accession number PRJNA1237765. The Whole Genome Shotgun project has been deposited at DDBJ/ENA/GenBank under the accession JBMFCY000000000.1. The raw sequencing reads have been deposited in the NCBI Sequence Read Archive (SRA) under accession number SRR33085572, while the corresponding genome assembly is available through GenBank under accession number GCA_049310985.1.
